# Intestinal malrotation associated with duodenal obstruction secondary to Ladd's bands

**DOI:** 10.1590/0100-3984.2015.0106

**Published:** 2016

**Authors:** Marco Aurélio Sousa Sala, Amanda Nogueira de Sá Gonçalves Ligabô, Mario Carlos Camacho de Arruda, João Maurício Canavezi Indiani, Marcelo Souto Nacif

**Affiliations:** 1 URC Diagnóstico por Imagem, São José dos Campos, SP, Brazil.; 2 Hospital ViValle, São José dos Campos, SP, Brazil.; 3 Universidade Federal Fluminense (UFF), Niterói, RJ, Brazil.

**Figure 1:**
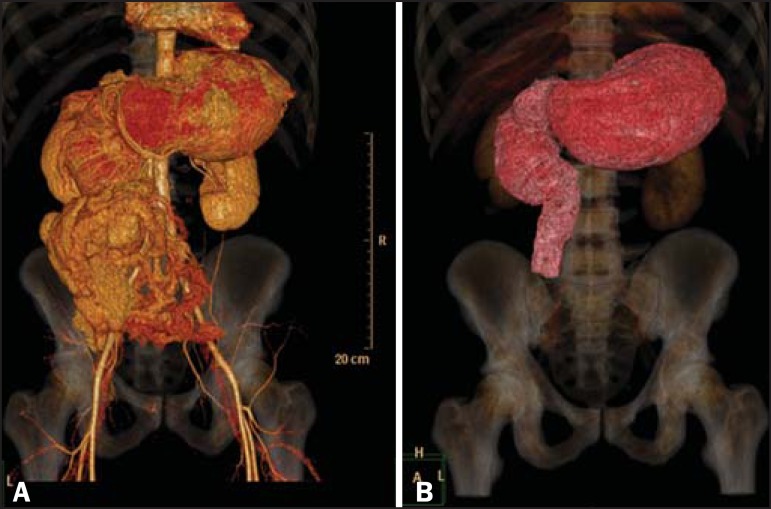
Multidetector computed tomography of the abdomen and pelvis, with three-dimensional reconstruction. Note the major fluid distension of the stomach and duodenum, with abrupt narrowing of the duodenal lumen at the transition from the second to the third portion of the duodenum **(A)**. The duodenal arch was short, with a vertical angle of Treitz and all of the loops shifted to the right, together with intestinal malrotation, the cecum and ascending colon appearing in the anterior and medial positions, occupying the mesogastrium **(B)**.

**Figure 2:**
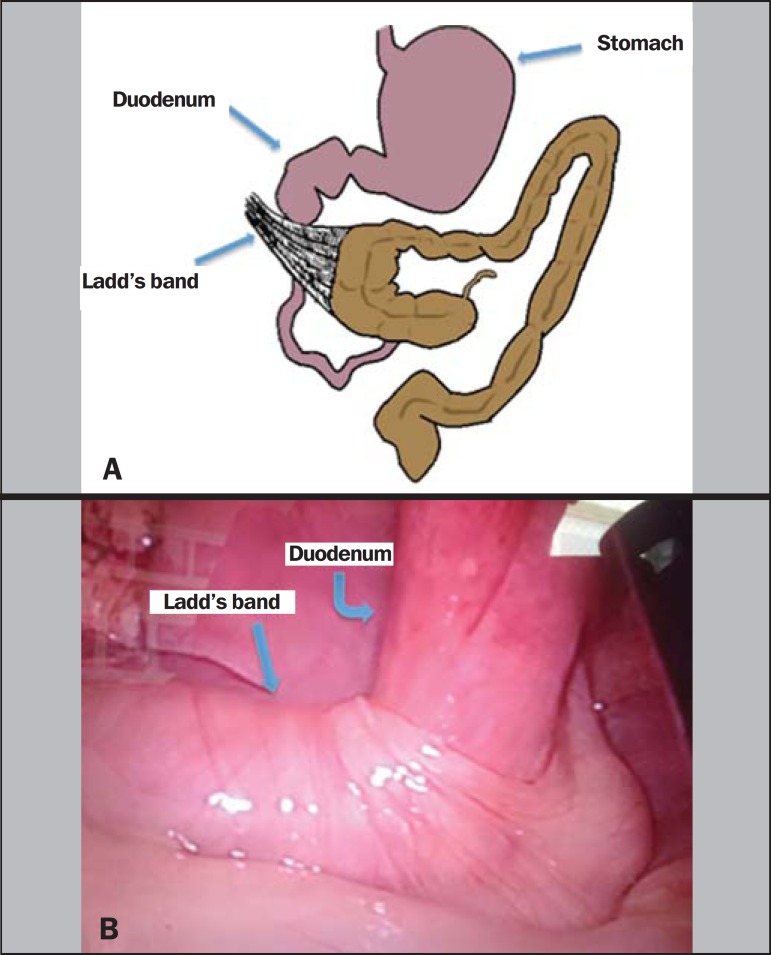
Classical depiction of malrotation with duodenal obstruction secondary to Ladd's bands **(A)**. The patient underwent laparoscopy (Ladd's procedure), which showed a Ladd's band **(B)**.

*Dear Editor*,

A 38-year-old male sought treatment in the emergency room, complaining of abdominal pain and bloating, accompanied by an inability to pass gas or eliminate feces. The patient underwent multidetector computed tomography of the abdomen and pelvis, with and without the administration of intravenous iodinated contrast media, which showed significant fluid distension of the stomach and duodenum, with abrupt narrowing of the duodenal lumen at the transition from the second to the third portion of the duodenum (Figure 1A). The duodenal arch was short, with a vertical angle of Treitz and all of the loops shifted to the right, together with intestinal malrotation, the cecum and ascending colon appearing in the anterior and medial positions, occupying the mesogastrium (Figure 1B). Those aspects are found in classical malrotation with duodenal obstruction secondary to Ladd's bands (Figure 2A). The patient underwent laparoscopy Ladd's procedure (Figure 2B) and was subsequently discharged in good condition, thereafter reporting no episodes of recurrence^(1,2)^.

The evaluation of the musculoskeletal system by imaging methods has been the subject of a number of recent studies in the radiology literature of Brazil^(3-9)^. Intestinal malrotation is a rare congenital condition, occurring in 1 out of every 200-500 live births. Most cases are diagnosed during the neonatal period, only 0.2% being diagnosed in adulthood. The condition can lead to chronic nonspecific symptoms in young adults, making it difficult to diagnose^(1,2,10)^.

Intestinal malrotation typically manifests as nonspecific abdominal discomfort, occasionally provoking abdominal pain related to obstruction of acute onset. Generally, the obstructions occur during the neonatal period and should be considered in all infants presenting with bilious vomiting and abdominal pain^(10,11)^.

The use of multidetector computed tomography in the emergency room has facilitated the diagnosis of malrotations, primarily in the context of congenital diseases that go undiagnosed until adulthood. This method, in addition to facilitating the evaluation of the loops, can aid in the assessment of the vasculature, which can be affected. Another important imaging method is radiological study with contrast, which can reveal a vertical duodenum and the absence of a duodenojejunal angle, as are observed in 80% of cases^(10,12)^.

The typical treatment for intestinal malrotation is Ladd's procedure, first described in 1936, which involves classical laparotomy. It is considered the gold-standard surgical treatment in cases of intestinal malrotation and can currently be performed safely by laparoscopy, as in the case presented here. The procedure consists in mobilization of the duodenum and right colon; the sectioning of adhesions (Ladd's bands, sometimes near the superior mesentery); and appendectomy. This aim of the treatment is to reduce the risk of acute-onset volvulus by placing the small intestine in a nonrotating position and broadening the base of the mesentery. Appendectomy is performed because of potential difficulty in diagnosing appendicitis in the future, given that the appendix would be far from the correct position^(1,11,12)^.

The diagnosis of intestinal malrotation associated with duodenal obstruction secondary to Ladd's bands should be considered in adult patients presenting with duodenal obstruction, a vertical duodenum, and malrotation of the small intestine with the cecum in the medial position. We believe that computed tomography is now the method of choice for the diagnosis of such malrotations.
